# International Society for Human and Animal Mycology (ISHAM)—New Initiatives

**DOI:** 10.3390/jof6030097

**Published:** 2020-06-30

**Authors:** Arunaloke Chakrabarti, Jacques F. Meis, Oliver A. Cornely

**Affiliations:** 1 Department of Medical Microbiology, Postgraduate Institute of Medical Education and Research, Chandigarh 160012, India; 2 Department of Medical Microbiology and Infectious Diseases, Canisius-Wilhelmina Hospital (CWZ), 6532 SZ Nijmegen, The Netherlands; jacques.meis@gmail.com; 3 Center of Expertise in Mycology Radboudumc/CWZ, 6532 SZ Nijmegen, The Netherlands; 4 Bioprocess Engineering and Biotechnology Graduate Program, Federal University of Paraná, Curitiba 81531-970, Brazil; 5 Department I of Internal Medicine, Excellence Center for Medical Mycology (ECMM), Medical Faculty and University Hospital Cologne, University of Cologne, 50937 Cologne, Germany; oliver.cornely@uk-koeln.de; 6 Cologne Excellence Cluster on Cellular Stress Responses in Aging-Associated Diseases (CECAD), University of Cologne, 50931 Cologne, Germany; 7 Zentrum fuer klinische Studien (ZKS) Köln, Clinical Trials Centre Cologne, University of Cologne, 50935 Cologne, Germany; 8 German Centre for Infection Research (DZIF), Partner Site Bonn-Cologne, Medical Faculty and University Hospital Cologne, University of Cologne, 50937 Cologne, Germany

**Keywords:** epidemiology, management, mycoses, diagnosis

## Abstract

Fungal infections have emerged as major threat to human beings. The world is not ready to face this formidable challenge due to limited awareness, insufficient laboratories, and difficulty in managing mycoses especially in developing countries. The International Society for Human and Animal Mycology (ISHAM) has undertaken several new initiatives to overcome these gaps, including a global outreach program with national affiliated mycology societies and other regional groups. ISHAM is working closely with the European Confederation of Medical Mycology (ECMM) and Global Action Fund for Fungal Infections (GAFFI) to enhance these efforts. The society has launched laboratory e-courses and is in the process of the development of clinical e-courses. ISHAM has partnered with regional conferences in South America and Asia by sponsoring international experts and young delegates. The society also supports young people from less developed countries to undergo training in laboratories of excellence. ISHAM facilitated the formation of the INFOCUS-Latin American Clinical Mycology Working Group (LATAM) and the Pan-African Mycology Working Group. The society appointed country ambassadors to facilitate coordination with national societies. Still, the task is enormous and ISHAM calls for strong advocacy and more coordinated activities to attract the attention of people from all disciplines to this neglected field.

## 1. Present Status

The emerging threat of fungal infections of plant, animal and man has been highlighted since the turn of this century [1,2]. The estimated fatalities due to fungal infections are around 1.5 million per year [3]. However, fungal infections were not appreciated by the majority of healthcare workers, authorities and funding agencies till the outbreak of *Candida auris* infection across the globe [4,5]. This outbreak drew the attention of the scientific community because of its similarity to bacterial outbreaks, with multi-drug resistance, rapid spread and multiple outbreaks with high mortality in hospital settings. In the latest CDC report on antimicrobial resistant threats, *C. auris* became the first fungal pathogen to be regarded as an urgent threat, side by side with carbapenem-resistant *Acinetobacter*, *Clostridioides difficile*, carbapenem-resistant Enterobacterales and drug-resistant *Neisseria gonorrhoeae* [6]. Moreover, this fungus is not easily identified in routine laboratory practices and requires specialized facilities like the availability of matrix-assisted laser desorption/ionization-time of flight (MALDI-TOF) equipment or nucleic acid sequencing [7].

The more formidable mycological challenges across the world are due to the following reasons:

- Lack of awareness of fungal diseases, especially in developing countries where two thirds of the world population lives. A recent survey in seven Asian countries reported a lack of formal training in medical mycology in 37% of clinicians; each clinician handles only 2–4 invasive fungal infections a month due to lack of suspicion and the absence of diagnostic mycology facilities; 80% of patients could not afford appropriate antifungal drugs. Clinicians fail to follow the standard guidelines of management; 34.3% of clinicians noted that they could not treat patients with appropriate antifungals due to the non-availability of the drug in their country [8];
- It is estimated that two million serious fungal infections are present at any time in Latin American countries, 1.7 million serious fungal infections in 15 African countries and more than two million serious fungal infections in eight countries in Southeast Asia [9];
- Certain game changers in fungal disease patterns have been noted over the years. Three important events over the last decade may explain the change in the paradigm of fungal infections.
  1. An outbreak of cryptococcosis due to *Cryptococcus gattii* in the temperate climate of Vancouver Island and the North-Western part of the United States of America, although the fungus had been known to be prevalent only in tropical climates. It was postulated that the fungus spread from Brazil to the temperate region by adaptation over the years [10,11,12];
  2. A growing outbreak of sporotrichosis due to *Sporothrix brasiliensis* is reported in Latin America, especially Brazil. Cat-to-man transmission is implicated for this outbreak, which is in contrast with the central dogma of endemic mycoses that occur from environment-to-man transmission [13,14];
  3. *Candida auris*, a fungus which behaves like bacteria. It is a serious threat due to antifungal resistance and rapid spread in healthcare facilities [15].
- Some of our common infections are posing difficulties in management and have become major public health issues, like the outbreak of tinea infections due to allylamine-resistant dermatophytes in some Asian countries [16,17,18,19];
- The limited number of diagnostic mycology laboratories in developing countries is the most important stumbling block in managing fungal infections;
  ○ A survey of eight countries in southeast Asia reported availability of non-culture-based diagnostics (beta-glucan, galactomannan, and *Histoplasma* antigen detection) in less than 25% of the laboratories [20];
  ○ A similar picture has been noted in Latin American and Caribbean countries in another survey [21];
  ○ Even in a developed country like the United Kingdom, a laboratory survey reported availability of β-glucan in 5%, galactomannan in 20%, and fungal PCR diagnosis in 6% laboratories only [22];
- Limited recognition of fungal diseases by international authorities is another barrier in the development of mycology;
  ○ Although after a prolonged advocacy, the World Health Organization (WHO) has agreed to include yeast antifungal resistance surveillance in the Global Antimicrobial Resistance Surveillance System (GLASS), the start of this surveillance is taking a long time due to inadequacies in diagnostic fungal laboratories and the evolving standard of antifungal resistance testing;
  ○ Mycetoma and chromoblastomycosis have been included in the WHO Neglected Tropical Disease (NTD) list, but many are still waiting to be included, like paracoccidioidomycosis, fungal keratitis and sporotrichosis;
  ○ Until the worldwide emergence of *C. auris* infections, fungal diseases were never included in public health challenge listings, though fungal keratitis, resistant dermatophyte infections, sporotrichosis, *Candida* vaginitis, etc., are major public health problems affecting large numbers of people. This requires concerted advocacy from organisations working in this field, including ISHAM;
- Antifungal drugs—Due to the limited scope of exploiting differences in metabolism between fungi and humans (both eukaryotes), few antifungal classes are in practice to treat human fungal infections. Both intrinsic resistance and emerging acquired resistance in antifungal drugs is further limiting the scope of antifungal management. Aggravating the problem, antifungal drugs are beyond the reach of the majority of the population in developing countries, either due to cost of the drugs or non-availability in certain countries. In a recent multi-centre study from India on mucormycosis, one quarter of the patients could not be treated with appropriate antifungal drugs despite timely diagnosis [23].

## 2. Targets of Mycology Societies

**Figure 1:**
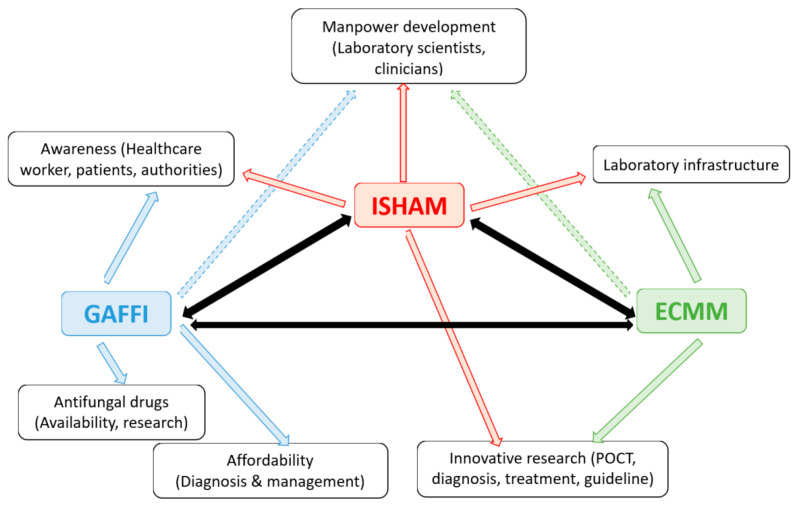
International Society for Human and Animal Mycology (ISHAM)’s close coordination with European Confederation of Medical Myology (ECMM) and Global Action Fund for Fungal Infections (GAFFI). Arrows indicate the activities and influence of different organizations in the indicated fields. Black arrows indicate close coordination of the three organizations.

Considering the above challenges, six major targets for improvement may be identified: (1) awareness among health care workers, patients and authorities; (2) laboratory infrastructure for diagnosis; (3) manpower development in this field; (4) research for innovative approaches in diagnosis and management, and relevant guidelines; (5) antifungal drugs and their availability; (6) affordability of diagnosis and therapy. The International Society for Human and Animal Mycology (ISHAM) has developed networking and global outreach with national affiliated societies (affiliation with ISHAM) and international societies to meet these challenges. Other than our society, two major players identified in this field are the European Confederation of Medical Mycology (ECMM), and Global Action Fund for Fungal Infections (GAFFI). To disseminate essential knowledge and provide training, perform epidemiologic and clinical studies and advocacy in the field of medical mycology for underdeveloped regions, ISHAM is working closely with both ECMM and GAFFI (Figure 1).

## 3. New Activities Initiated by ISHAM

### To Achieve the Above Targets ISHAM Initiated the Following Activities

**ISHAM laboratory e-course:** ISHAM launched a laboratory e-course of 12 modules with the help of Dr. Ruth Ashbee. The course includes an overview of fungal diseases and antifungals, diagnostic methods, and taxonomy. Each of the diagnostic methods is then covered in detail in individual modules which include the identification of yeast and mould, direct microscopy, culture and histopathology, antigen and antibody detection, molecular diagnosis, antifungal susceptibility testing, and approaches in diagnosis in different patient populations. The course is being accredited by the European Accreditation Council for Continuing Medical Education (EACCME). Participants who pass the final course examination receive a certificate from ISHAM. This course is free for ISHAM members and is nominally charged for non-ISHAM members. The course may be accessed at https://isham.scholarlms.com/;

**ISHAM clinical e-course:** Similar to the laboratory e-course, a clinical e-course is in preparation under the leadership of Dr. John Perfect. Three eminent clinicians, Dr. Martin Hoenigl, Dr. Ilan Schwartz, and Dr. Matteo Bassetti, are working on this task. The course will follow the concept of 12 clinical mycological cases discussing epidemiology, diagnosis, and management;

**Partnering with regional conferences:** International conferences for ISHAM are held with a gap of three years and large numbers of young scientists/doctors fail to attend the 3-yearly ISHAM conferences due to insufficient funding support. ISHAM desires to reach to them in their region. The society has started partnering with regional societies or working groups during those two-year gap periods to organize regional conferences. In this partnership, ISHAM sponsors international leading experts as faculty of the conference and sponsors young society members to attend the conference. The society also sponsors the venue and audio-visual facilities for the conference. The society has already partnered the 17th INFOCUS held in Salvador, Brazil during 14–16 November 2019 and has proposed to partner with the Asian Fungal Working Group (AFWG) congress in Bangkok, Thailand during 5–7 August 2021. In Salvador, the delegate number increased compared to previous years, especially among young delegates who were able to interact with international experts and showcase their research in front of them;

ISHAM is continuing the scientific support for regional meetings held in the Middle East. This is called Integrated Networking Forum for Research in Mycology (INFORM). The scientific programme is developed by ISHAM, and Gilead Sciences is taking care of the organization;

**Development of Regional Working Groups:** ISHAM is stimulating development and training in medical mycology in those areas of the world where awareness is lacking, and laboratory facilities are limited. With the success of the ISHAM Asian Fungal Working Group (functioning in nine countries in Asia for education and training and multi-centre research for the last 11 years), ISHAM has facilitated the formation of two new regional working groups (a) INFOCUS-Latin American Clinical Mycology Working Group (LATAM), (b) Pan African Mycology Working Group. These two working groups will stimulate the improvement of medical mycology in Latin America and Africa, respectively;

**ISHAM country ambassadors:** ISHAM has appointed an ambassador in each country, who would work as interface between ISHAM and national mycology societies. This would help in coordinating activities in that country;

**Young ISHAM:** Young ISHAM is a separate working group within ISHAM that has fulfilled an essential function for the last seven years. One of the vice-presidents of the council is dedicated to providing leadership for this working group. The young (below 40 years) doctors/researchers get the opportunity to become a society member at reduced rates. The ISHAM Vice-President is organizing regional Young ISHAM groups of 3–5 people from different continents to identify mycology needs of beginners in the field in different parts of the world. To restructure Young ISHAM, the council has decided to select at least two young regional ambassadors, one in basic mycology and another one from clinical mycology. Two training fellowships every year will be sponsored. The young ISHAM group conducts activities that are beneficial for young members in education, placement and coordinated international activities;

**ISHAM funding:** ISHAM funds working groups (30 working groups function under the society) to run meetings or courses; supports organizers of specialist medical mycology conferences, workshops or training courses; promotes Professor-in Residence programs to run courses in developing countries; supports young ISHAM training fellowships (this year ISHAM supported three African students and one Iranian student); and also supports the young ISHAM group by providing investigator awards;

**ISHAM journals:** The Society’s two academic journals, *Medical Mycology* and *Medical Mycology Case Reports*, continue to grow and provide a platform for ground-breaking research and clinical experience for a global readership. *Medical Mycology Case Report* is an open-access journal. The other journal, *Medical Mycology* will become online publication only from 2021 onwards

Despite all these new initiatives, we recognize the enormous tasks which lie ahead, as large numbers of people in developing countries are vulnerable to fungal infections. We need a strong advocacy to move the administrators in each country to recognize the seriousness of the impending fungal problem and to take appropriate measures. We vow that ISHAM will work relentlessly to achieve this target.
